# Desmin common mutation is associated with multi-systemic disease manifestations and depletion of mitochondria and mitochondrial DNA

**DOI:** 10.3389/fgene.2015.00199

**Published:** 2015-06-05

**Authors:** Elizabeth M. McCormick, Lawrence Kenyon, Marni J. Falk

**Affiliations:** ^1^Division of Human Genetics, Department of Pediatrics, The Children's Hospital of PhiladelphiaPhiladelphia, PA, USA; ^2^Department of Pathology, Thomas Jefferson University HospitalPhiladelphia, PA, USA; ^3^Department of Pediatrics, University of Pennsylvania Perelman School of MedicinePhiladelphia, PA, USA

**Keywords:** desmin-related myopathy, *DES*, mitochondrial disease, whole exome sequencing, mitochondrial DNA depletion

## Abstract

Desmin (*DES*) is a major muscle scaffolding protein that also functions to anchor mitochondria. Pathogenic *DES* mutations, however, have not previously been recognized as a cause of multi-systemic mitochondrial disease. Here, we describe a 45-year-old man who presented to The Children's Hospital of Philadelphia Mitochondrial-Genetics Diagnostic Clinic for evaluation of progressive cardiac, neuromuscular, gastrointestinal, and mood disorders. Muscle biopsy at age 45 was remarkable for cytoplasmic bodies, as well as ragged red fibers and SDH positive/COX negative fibers that were suggestive of a mitochondrial myopathy. Muscle also showed significant reductions in mitochondrial content (16% of control mean for citrate synthase activity) and mitochondrial DNA (35% of control mean). His family history was significant for cardiac conduction defects and myopathy in multiple maternal relatives. Multiple single gene and panel-based sequencing studies were unrevealing. Whole exome sequencing identified a known pathogenic p.S13F mutation in *DES* that had previously been associated with desmin-related myopathy. Desmin-related myopathy is an autosomal dominant disorder characterized by right ventricular hypertrophic cardiomyopathy, myopathy, and arrhythmias. However, neuropathy, gastrointestinal dysfunction, and depletion of both mitochondria and mitochondrial DNA have not previously been widely recognized in this disorder. Recognition that mitochondrial dysfunction occurs in desmin-related myopathy clarifies the basis for the multi-systemic manifestations, as are typical of primary mitochondrial disorders. Understanding the mitochondrial pathophysiology of desmin-related myopathy highlights the possibility of new therapies for this otherwise untreatable and often fatal class of disease. We postulate that drug treatments aimed at improving mitochondrial biogenesis or reducing oxidative stress may be effective therapies to ameliorate the effects of desmin-related disease.

## Introduction

A 45-year-old Caucasian man presented to The Children's Hospital of Philadelphia Mitochondrial-Genetics Diagnostic Clinic for evaluation of suspected mitochondrial disease based on arrhythmia, cardiomyopathy, progressive lower extremity myopathy, and peripheral neuropathy. His cardiac problems presented as episodic dizziness and congenital heart block that were noted in his late 20 s, with a pacemaker placed at 30 years of age. Over the next decade, he developed intermittent atrial flutter, right hypertrophic cardiomyopathy, and congestive heart failure. Myopathic symptoms began in his mid-late 30 s with leg weakness that progressed following pneumonia at 38 years of age. By age 45, he was no longer able to run and had significant difficulty ambulating. Electromyogram performed at that time revealed a distal myopathy with myotonic discharges and mild length-dependent neuropathy. A detailed review of systems was significant for lifelong short stature, progressive photophobia, hoarse voice, gastroesophageal reflux, swallowing dysfunction, progressive truncal obesity, progressive bloating, anger, and intermittent depression. Review of prior laboratory screening studies were significant for an elevated GGT, elevated indirect bilirubin, and elevated creatine kinase (315 U/L). Prior genetic testing only included *DM1* and *DM2* expansion analyses, which were normal.

**Figure 1:**
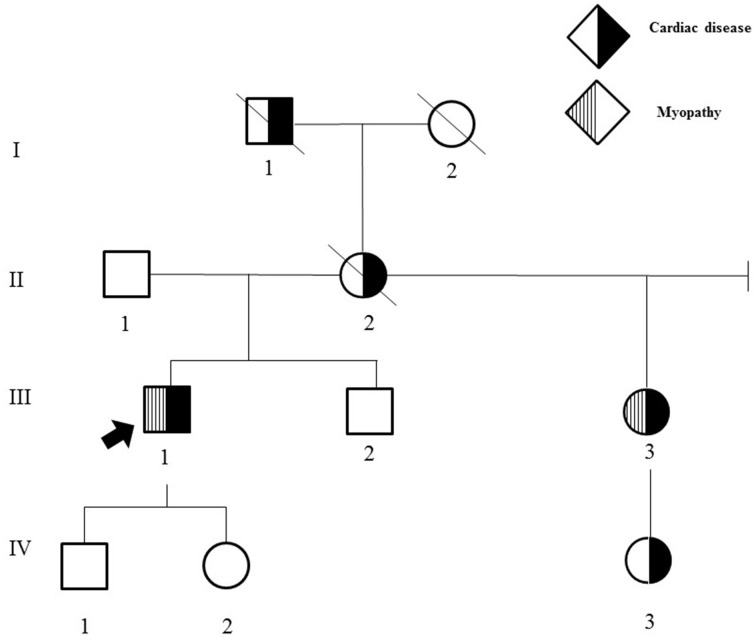
**Family history**. II-2 had pacemaker placement at 38 years of age and sudden cardiac death at age 62. III-3 had dizziness and syncope onset at age 30, cardiac conduction block requiring pacemaker placement at age 30, extremity weakness and calf muscle wasting onset in the mid-50 s; choking episodes beginning in the 50 s, and hand numbness and tingling onset in the late 20 s that largely resolved following carpal tunnel surgery. In addition, she developed obesity, photophobia, a progressively enlarging palatal cyst protruding to her right nostril resulting in limited sense of smell at age 30, difficulty hearing with background noise in her mid-50 s; and irritable bowel syndrome with alternating constipation and diarrhea in her early 50 s. III-2 has intellectual disability and autism. I-1 died at age 39 from presumed sudden cardiac death, had a history of “dropsy” with recurrent syncope, edema, and dizzy spells. I-2 died at age 92 years from cancer and required pacemaker placement in her 70 s. IV-3 had tachycardia requiring ablation at 10 years of age.

His family history was significant for multiple individuals with cardiac and/or neuromuscular disease (Figure 1).

The proband's physical examination at age 45 was significant for short stature, distant heart sounds, abdominal obesity, small hands, and cold and purplish-colored feet. He was unable to get onto the exam table without assistance, but could lie back and sit up with minimal assistance. He had profound thigh weakness and was unable to lift either thigh even slightly without resistance. He had normal 5/5 strength of the upper extremities, 4/5 strength in the fingers, and 3–4/5 strength in the lower legs and feet with significant calf muscle wasting. He had 1+ biceps and patellar deep tendon reflexes, but ankle reflexes could not be elicited. He had a slapping gait, was unable to perform toe or heel walking, and had difficulty performing tandem gait. He was unable to stoop at the knees and recover. He reported sharp and dull sensation on his feet and legs.

Clinical follow-up at age 46 was notable for interval development of progressive leg muscle weakness and tingling, especially of the calves, progressive atrophy of arm and leg muscles, increased leg swelling, and a constant cold sensation of his legs. Physical examination revealed cervical kyphosis and 2+ pitting edema of the legs with swollen ankles obscuring all landmarks. He no longer could sit up with minimal assistance but instead needed to roll onto his side to get from a supine to a sitting position. Upper extremity strength was stable. He reported an inability to feel the floor with his feet and now had only minimal control of foot and ankle movements. Patellar and ankle DTRs could not be elicited.

Metabolic laboratory studies performed at age 45 were normal, including a comprehensive chemistry panel, complete blood count, lipoprotein profile, thyroid function screen, plasma and urine amino acid quantitative analyses, pyruvate, lactate, ammonia, carnitine analysis, acylcarnitine profile, urinalysis, and urine organic acid analysis, with the only exception being a mildly elevated creatine kinase activity (258 U/L, normal 55–170 U/L).

**Figure 2:**
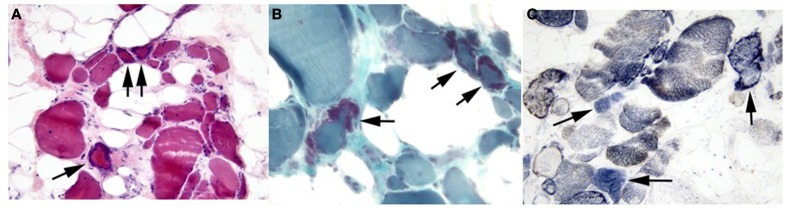
**Proband's skeletal muscle cryostat sections**. Muscle sections stained with **(A)** H&E or **(B)** modified Gomori trichrome stain demonstrate marked variability in fiber size, with fiber atrophy and fatty infiltration. Ragged red fibers are marked by arrows in both panels. **(C)** SDH/COX staining demonstrated several fibers (arrow) with SDH activity (blue) that lack COX activity (brown). Original magnifications: **(A)**, 200X; **(B,C)**, 400X.

Left quadriceps muscle biopsy was performed at age 45, which revealed generalized muscle fiber atrophy with fatty-fibrous tissue infiltration. Ragged red fibers and SDH positive/COX negative fibers consistent with a mitochondrial myopathy were identified (Figure 2). No ultrastructural mitochondrial morphologic abnormalities were evident by electron microscopy although two cytoplasmic bodies were present.

Muscle mitochondrial DNA content by quantitative PCR analysis revealed mitochondrial DNA depletion, at 35% of the mean value of age- and tissue-matched controls. Per Baylor Medical Genetics Clinical Diagnostic Laboratory standard techniques, mitochondrial and nuclear DNA contents are determined by real-time quantitative PCR using specific primers for the mitochondrial tRNALeu(UUR) gene and the nuclear ß-2-microglobulin (ß2M) gene (Bai et al., 2004; Bai and Wong, 2005; Dimmock et al., 2008, 2010; Wong et al., 2008; Venegas et al., 2011). The mitochondrial DNA copy number is calculated from the difference in threshold cycle numbers of mitochondrial DNA and nuclear DNA. Control samples were obtained from over 300 individuals suspected of having mitochondrial disease but mutations in genes known to cause mitochondrial DNA depletion were not identified.

Muscle mitochondrial DNA sequencing by next-generation sequencing did not detect large deletions or known deleterious point mutations.

Muscle mitochondrial respiratory chain enzyme activity analyses revealed significant deficiency of overall mitochondrial content as evidenced by citrate synthase activity reduced to 16% of the control mean; relative deficiency of several electron transport chain enzyme activities was also present that largely normalized (complex II+III) or improved to >30% of control (complex II, complex IV) when corrected for the low citrate synthase activity (Table 1). This reduction after correction for citrate synthase may suggest that, in addition to reduced mitochondrial mass, the energetic efficiency may be compromised. Per Baylor Medical Genetics Clinical Diagnostic Laboratory standard technique, the electron transport chain enzymes were assayed at 30°C using a temperature-controlled spectrophotometer. The activities of complex I, complex II, complex I+III, complex II+III, and complex IV were each measured in duplicate using different electron acceptors/donors. The increase or decrease of cytochrome c at 550 nm was measured for complex I+III, II+III, or complex IV. The activity of complex I was measured by following the oxidation of NADH at 340 nm. For complex II, the reduction of 2,6-dichloraindophenol (DCIP) at 600 nm was measured. Enzyme activities were normalized against citrate synthase activity (Ye and Hoppel, 2013; Vigelsø et al., 2014) as citrate synthase was greater than one standard deviation above or below control mean (Trounce et al., 1996; Walker et al., 1996; Bernier et al., 2002; Gellerich et al., 2004; Enns et al., 2005; Kirby et al., 2007). The 95% confidence interval is used as control, with several hundred individuals included in each control range. Additionally, a concurrent mouse muscle wild type control is analyzed with each enzymologic assay for quality control.

**Table 1:** **Muscle mitochondrial respiratory chain enzyme activity analyses**.

**Electron transport chain activities**	**ETC complexes**	**Percent of mean (before correction for decreased citrate synthase) (%)**	**Percent of mean (after correction for decreased citrate synthase) (%)**	**Patient activity (nmol/min/mg protein)**	**Control ± standard deviation (nmol/min/mg protein)**
NADH:cytochrome c reductase (rotenone sensitive)	Complex I+III	0	0	0	9.1 ± 2.5
Succinate dehydrogenase	Complex II	6	40	0.51	8.11 ± 2.44
Succinate:cytochrome c reductase	Complex II+III	18	115	0.87	4.9 ± 1.1
Cytochrome c oxidase	Complex IV	6	39	1.8	29.2 ± 9.1
Citrate synthase		16	100	44	280 ± 95

Genetic testing was performed in sequential fashion. At age 45, blood-based mitochondrial DNA sequencing by next-generation sequencing analysis and frataxin gene expansion analysis were normal. Following muscle biopsy studies, *LMNA* sequencing was recommended and was normal, as was sequencing, deletion, and duplication analysis of 101 nuclear genes known to be associated with mitochondrial disease. Whole exome sequencing (WES) on a clinical diagnostic basis at Baylor Medical Genetics Laboratory was then recommended, although no parental samples were available for analysis. WES identified a known mutation in the *DES* gene, namely c.38C>T;p.S13F, that was previously associated with desmin-related myopathy. Specifically, the p.S13F mutation had previously been reported as a disease-causing founder mutation in Dutch patients reported to have skeletal muscle weakness and cardiac involvement (Bergman et al., 2007; van Spaendonck-Zwarts et al., 2012). Targeted p.S13F mutation analysis in his symptomatic, maternal half-sister was positive and negative in his niece with a history of tachycardia requiring ablation at 10 years of age. The family is considering targeted mutation analysis in proband's full brother and children.

## Background

Desmin-related myopathy caused by missense mutations in *DES* has been recognized to be a fully penetrant, autosomal dominant condition with variable manifestations that largely include myopathy, cardiomyopathy, and arrhythmias (Goldfarb et al., 1998; Dalakas et al., 2000; Bergman et al., 2007; van Spaendonck-Zwarts et al., 2012). Desmin expression is highest in heart, followed by muscle, and gastrointestinal tract^1^. However, there is also expression known in the peripheral nerve and reproductive organs including cervix, prostate, and uterus. The p.S13F mutation identified in the family described here is a previously identified, well-characterized pathogenic alteration and was first recognized as a disease-causing founder mutation in Dutch patients with varying degrees of skeletal muscle weakness and cardiac involvement (Dalakas et al., 2000). Prior phenotypic analysis of individuals carrying the p.S13F mutation in *DES* found that all individuals had cardiac conduction disease and/or cardiomyopathy while neuromuscular features were not present in everyone (van Spaendonck-Zwarts et al., 2012). Both the proband and his maternal half-sister in the present family had cardiac and neuromuscular involvement, both of which were more severe and progressed at an earlier age in the proband, as well as gastrointestinal dysfunction in both and progressive neuropathy and mood disorder in the proband. Our proband also had two cytoplasmic bodies identified on his muscle biopsy histology, which are not specific but can be seen in desmin-related myopathies. While the proband's niece with a history of tachycardia in childhood did not harbor the familial *DES* mutation, there are many recognized non-inherited causes of arrhythmia that could have been the source in this individual.

## Discussion

While the cardiac and neuromuscular phenotype of desmin-related myopathy is well characterized, there is scarce reported literature on desmin-related myopathies being associated with mitochondrial dysfunction or disease. Desmin (*DES*) is a key scaffolding protein found in muscles that is known to function as a mitochondrial anchor (Stromer and Bendayan, 1988). Prior studies in desmin-null mice revealed subsarcolemmal accumulations of mitochondria in muscle and overall increased numbers of mitochondria, as well as impaired mitochondrial respiratory capacity (Milner et al., 2000). One previous case report in a man with a frameshift *DES* mutation (c.5141_5143insA; p.K239fsX242) revealed clustering of muscle mitochondria, normal respiratory chain enzymology in skeletal muscle homogenates, and decreased maximal rates of respiration and complex I in isolated saponin-permeabilized skeletal muscle fibers (Schroder et al., 2003). However, there was no report of decreased mitochondrial mass or mitochondrial DNA content either in that case or more generally in desmin-related myopathy. Additionally, two siblings with progressive muscle fatigue and weakness were found to be compound heterozygous for mutations in *DES* with carrier status confirmed in both asymptomatic parents. Muscle biopsy in one of these siblings revealed myopathic features, 5% COX deficient fibers on immunohistochemical analysis, and absent desmin staining. No mitochondrial DNA sequencing, mitochondrial DNA content analysis, nor enzymatic activity assays of mitochondrial respiratory chain function was reported. Electron microscopy showed no structural abnormalities in the mitochondria although there was variability noted in their size and distribution (Henderson et al., 2013).

Additionally, while only muscle mitochondrial DNA content was assessed in this proband, it is not unreasonable to postulate that his multisystemic involvement can be due to muscle and/or peripheral nerve involvement. Indeed, neurogenic disease and/or polyneuropathy was reported on electromyogram analysis of at least three other individuals with *DES* mutations, including one confirmed to have the p.S13F mutation (Bergman et al., 2007).

## Concluding remarks

We report here a case of progressive cardiac and neuromuscular disease that was clinically suspected as a mitochondrial disorder based on his progressive, multi-systemic involvement including neurologic, gastrointestinal, and mood manifestations. Muscle biopsy in this case revealed ragged red fibers and SDH positive/COX negative fibers, findings consistent with classical primary mitochondrial disease, as well as pronounced depletion of mitochondrial DNA content and reduced mitochondrial mass, and complex I deficiency. Numerous single-gene and panel-based molecular analyses were unrevealing. Clinically-based whole exome sequencing revealed the underlying etiology in the proband and his similarly affected sister to be a previously reported pathogenic mutation in the *DES* gene. This case expands the phenotype of desmin-related myopathy to include clinical features in the nervous and gastroenterologic systems, as are typical of mitochondrial disease. Recognizing that significant mitochondrial depletion occurs in desmin-related myopathy is especially important to enable development of new therapeutic avenues for this progressive and often fatal disease, such as potentially through stimulation of mitochondrial biogenesis or reduction of oxidative stress that commonly occurs in mitochondrial disorders (Parikh et al., 2009). Finally, these data are suggestive that there may exist other neuromuscular diseases caused by mutations in structural proteins that interact directly with mitochondria.

### Conflict of interest statement

The authors declare that the research was conducted in the absence of any commercial or financial relationships that could be construed as a potential conflict of interest.

## Footnotes

^1^GeneHub-GEPIS. Available online at: http://research-public.gene.com/cgi-bin/genentech/genehub-gepis/web_search-result.pl?id=GENE2507&CHR=CHR2&CHR_END=219999704&CHR_START=219991342&DESCT=desmin&END=468412689&GENE_NAME=DES&ID=GENE2507&SPECIES=HUMAN&START=468404327&STRAND=1&intype=1&xrefid=DES&species=human
